# Histomorphometry of the Sural Nerve for Use as a CFNG in Facial Reanimation Procedures

**DOI:** 10.3390/jcm12144627

**Published:** 2023-07-12

**Authors:** Andreas Kehrer, Katharina S. Hollmann, Silvan M. Klein, Alexandra M. Anker, Ernst R. Tamm, Lukas Prantl, Simon Engelmann, Samuel Knoedler, Leonard Knoedler, Marc Ruewe

**Affiliations:** 1 Department of Plastic, Hand, and Reconstructive Surgery, University Hospital Regensburg, 93053 Regensburg, Germany; 2 Division of Plastic and Facial Palsy Surgery, Hospital Ingolstadt, 85049 Ingolstadt, Germany; 3 Department of Molecular Pathology, Massachusetts General Hospital, Harvard Medical School, Boston, MA 02115, USA; 4 Department of Human Anatomy and Embryology, University of Regensburg, 93053 Regensburg, Germany; 5 Department of Plastic Surgery and Hand Surgery, Klinikum Rechts der Isar, Technical University of Munich, 81675 Munich, Germany

**Keywords:** axons, axons/analysis, dissection, facial injuries/surgery, facial muscles/innervation, facial nerve/anatomy and histology, humans, nerve fibers, facial paralysis, reconstructive surgical procedures/anatomy and histology

## Abstract

Facial palsy (FP) is a debilitating nerve pathology. Cross Face Nerve Grafting (CFNG) describes a surgical technique that uses nerve grafts to reanimate the paralyzed face. The sural nerve has been shown to be a reliable nerve graft with little donor side morbidity. Therefore, we aimed to investigate the microanatomy of the sural nerve. Biopsies were obtained from 15 FP patients who underwent CFNG using sural nerve grafts. Histological cross-sections were fixated, stained with PPD, and digitized. Histomorphometry and a validated software-based axon quantification were conducted. The median age of the operated patients was 37 years (5–62 years). There was a significant difference in axonal capacity decrease towards the periphery when comparing proximal vs. distal biopsies (*p* = 0.047), while the side of nerve harvest showed no significant differences in nerve caliber (proximal *p* = 0.253, distal *p* = 0.506) and axonal capacity for proximal and distal biopsies (proximal *p* = 0.414, distal *p* = 0.922). Age did not correlate with axonal capacity (proximal: R = −0.201, *p* = 0.603; distal: R = 0.317, *p* = 0.292). These novel insights into the microanatomy of the sural nerve may help refine CFNG techniques and individualize FP patient treatment plans, ultimately improving overall patient outcomes.

## 1. Introduction

**Figure 1:**
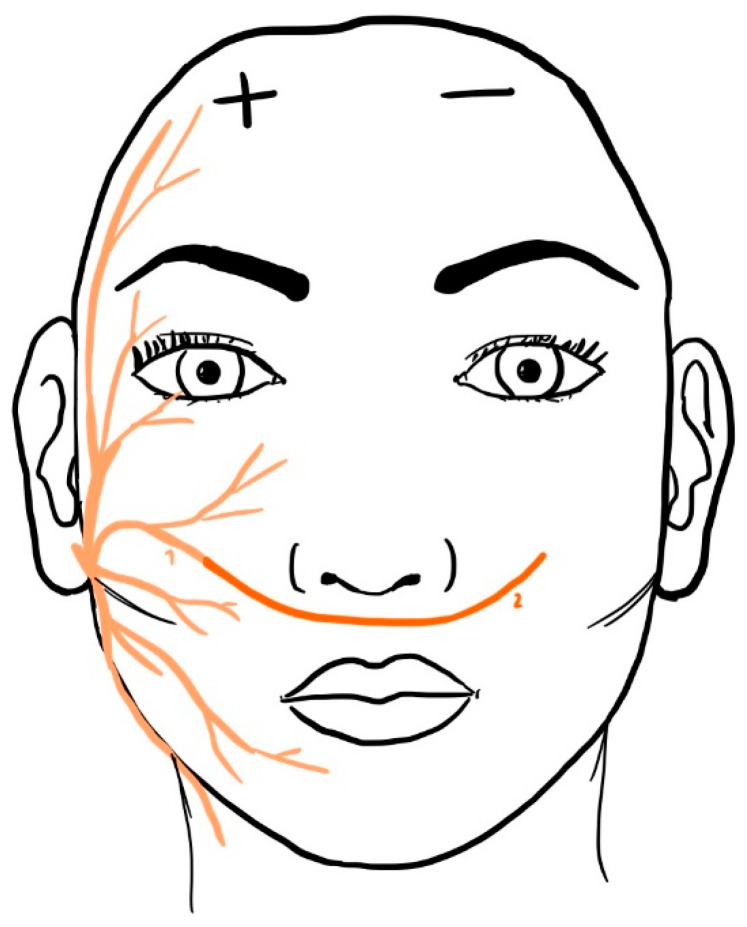
This schematic drawing represents a CFNG procedure in which the sural nerve graft is coaptated to a facial branch and tunneled through the face to the paretic side. On the paretic side, it can reinnervate facial muscles directly (nerve-to-muscle) or indirectly (nerve-to-nerve) in the early stages of facial palsy. When the mimic muscles on the paretic side have atrophied in late cases of FP, a second stage free muscle flap is needed. The CFNG is then coapted to the corresponding nerve, usually the obturator nerve of the gracilis muscle.

Patients with facial palsy (FP) suffer both functionally and esthetically due to a loss of facial muscle function [1,2,3,4]. Facial reanimation surgery aims to increase quality of life for patients by restoring muscle function and symmetry. This has been shown to have a positive impact on patients’ mental health showing significantly more joy and less negative emotion [5]. Cross Face Nerve Grafting (CFNG) characterizes a surgical procedure using nerve grafts to redirect intact donor nerve fibers across the face for reanimating contralateral target muscles in the paralyzed facial half (Figure 1). It is employed as either an augmentative procedure for reinnervating weakened facial muscles in early cases of FP or linked to a second stage muscle free flap in late cases of FP, where the mimic muscles have atrophied [6,7,8,9,10,11]. Most frequently, the gracilis muscle is used and its associated obturator nerve branch will be coapted to the CFNG [12,13]. For further treatment, especially second stage muscle free flap following CFNG, knowledge about the different stages of nerve regeneration is crucial for optimal timing and outcomes [14,15]. The second stage procedure can usually be applied after 9 to 12 months following CFNG, as determined by the nerve regeneration rate [15,16]. Nerve regeneration in the peripheral nervous system is a multi-staged process in which damaged or severed peripheral nerves repair, outgrow, and reconnect when aiming for functional restoration. Initially, after nerve injury—also referring to cut-off for grafting—a degenerative process, known as Wallerian degeneration, is initiated. Schwann cells (SC), which provide a scaffold and functional support for nerve fibers, orchestrate a growth-enhancing milieu for axonal outgrowth and form structural guidance channels for regenerating axons. Axonal sprouting occurs as new fibers, called growth cones, extend from the proximal stump of the damaged nerve, guided by various chemical signals including neurotrophic factors. As the growth cones reach the other side of the nerve trunk, they encounter another barrier referred to as the distal stump. Here, the reconnection of nerve fibers requires additional growth factors and adhesion molecules. Functional restoration is achieved when the regenerating axons reestablish connection with their targeted tissues [17,18,19].

For bridging the distance from the non-paralyzed to the paralyzed facial side a nerve graft with great length is required. The sural nerve represents a reliable nerve graft with little donor side morbidity and can provide 30–40+ centimeters in length [20].

Sprouting axons must cross two coaptation sites and a long reinnervation distance in CFNGs; therefore, only a fraction of the original donor axon count may reach the target organ. Different studies in the literature report on the outcomes and optimization of reinnervation in CFNG procedures [21,22]. Ultimately, the axons of the nerve graft perish in Wallerian degeneration, but it is likely that the sheath structures remain intact and can act as a scaffold for sprouting. While the macroanatomy of the sural nerve is well described, little attention has been paid to microanatomic features [23]. Determining the axon count is crucial for individualizing and optimizing CFNG. Matching the cross-sectional diameter and axonal capacity of the donor nerve, the nerve graft and recipient nerve might improve regeneration. Exact axon counts are important for successful reinnervation and functional outcomes. This line of research is important to improve postoperative outcomes and patient care [24,25,26,27].

The aim of our study was to investigate the microanatomy of the sural nerve, which is harvested during CFNG procedures. Particular attention was paid to fascicular structure, nerve branch diameter, and axonal capacity.

## 2. Materials and Methods

Between January 2020 to December 2021, 15 patients undergoing FP reconstruction with use of sural nerve grafts in terms of CFNG were recruited for this study (Figure 1). Patients were instructed preoperatively for biopsy of the sural nerve graft and gave written informed consent for this study. The study was approved by the Institutional Review Board Committee of the University of Regensburg (Reference number: 20-2081-101) and was designed in accordance with the Declaration of Helsinki.

### 2.1. Sural Nerve Harvest

Standardized harvest of the sural nerve was conducted under 4×-loupe magnification utilizing microsurgical instruments and a nerve stripper (Assmus nerve striper, Aeskulap, B. B. Braun Melsungen AG, 34212 Melsungen, Germany) [20]. The sural nerve was identified distally at the midpoint between the lateral malleolus and Achilles tendon, using a 3-cm vertical incision. The nerve is found adjacent and profound to the lesser saphenous vein and was dissected free from the surrounding tissue. The sural nerve was neurolyzed distally and was sharply dissected. The ring of the nerve harvester was then guided over the end of the nerve and advanced proximally. Resistance may occur at the level of the junction of the middle and distal thirds of the leg. This usually represents the location of the anastomosis between the lateral and medial sural cutaneous nerves [28]. On this point, which was mostly found 16 cm proximally to the lateral malleolus, the nerve pierces the gastrocnemius fascia and ends its epifascial course. To avoid tearing the nerve while harvesting, it is recommended to place the incision there [20]. The harvester was advanced proximally to the popliteal fossa, where the sural nerve was cut and retracted.

### 2.2. Biopsy Sampling, Histological Processing, and Digitalization

The biopsies were obtained at the distal and proximal end of the nerve graft under the surgical microscope (Zeiss Kinevo, Meditec AG, Oberkochen, Germany). A modified EM-fixation solution as described by Ito and Karnovsky (2.5 percent PFA; 2.5 percent glutaraldehyde) was used to fixate the nerve graft [29]. The samples collected in the operating room were taken to the laboratory in a refrigerated transport box. In the laboratory, the samples were processed according to the standard of our work group [24]. The probes were rinsed with 0.1 M natriumcacodylat buffer and osmium ferrocyanide was added for secondary fixation. Afterwards, the biopsies were rinsed with purified water prior to gradual dehydration with EtOH and Acetone. Following the embedment in Epoxy Resin (EPON; HexionSpecialty Chemicals, Inc., Columbus, OH, USA) and incubation at 60 degrees Celsius, semi-thin sections (1 µm) were cut with the ultramicrotome (LKB, Sollentuna, Sweden) and stained with PPD (p-phenylenediamine). Finally, the samples were assessed using a digital microscope (Zeiss, Axio Imager Z1 with Axio Cam MR and Zeiss ZEN computer software). Photographs were obtained at 3200 K. Grey color images were recorded at 200× magnification.

### 2.3. Axonal Quantification and Diameter Measurement

**Figure 2:**
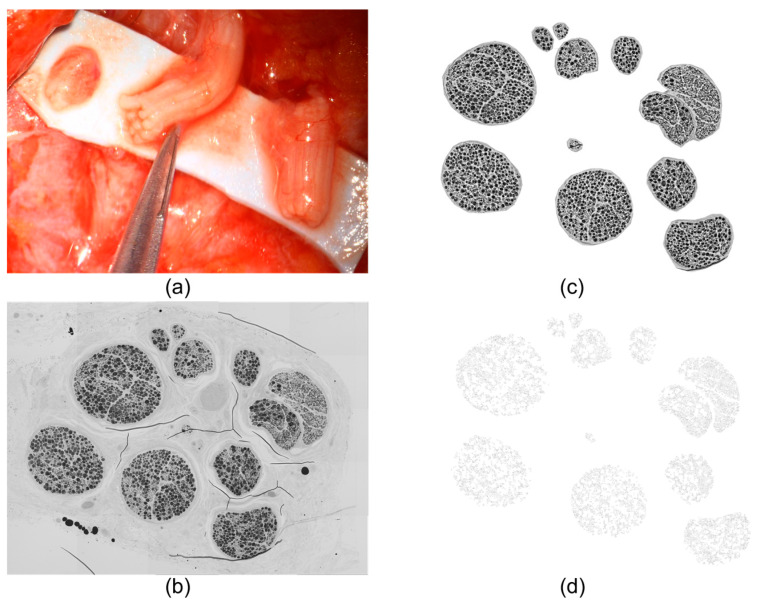
This figure illustrates the processing of the nerve biopsies. The biopsies are obtained under a surgical microscope (Zeiss Kinevo, Meditec AG, Oberkochen, Germany) with fine microsurgical instruments (**a**). After digitalization of the fixated and stained cross-sections (**b**) semi-automated axon quantification is carried out (**c**,**d**), resulting in 9201 and a cross-sectional diameter of 2.39 mm.

The distinct working steps of determining axonal capacity and cross-sectional nerve diameter are illustrated in Figure 2. We performed a semi-automated axonal quantification developed by our workgroup for Fiji freeware, while fascicles were counted manually [24]. Diameter measurement of the cross-sections was conducted with a digital microscope at 2.5× magnification. The nerve cross-section was measured via two orthogonal vectors. For this measurement, we included the epineurium, vasa nervorum, and surrounding fat tissue. Assuming a round or oval-shaped nerve structure, we calculated the cross-sectional nerve diameter as the mean of the two vectors.

### 2.4. Inclusion Criteria and Statistical Analysis

We defined the following quality standards for sample inclusion: integrity of the neural structure and its surrounding tissue, staining of the complete cross-section area, full exposure of the section, and entirely orthogonal cuts showing a clear image of the axons.

Statistical analysis was performed with SPSS (IBM SPSS Statistics for Windows, Version 27.0.1 Armonk, NY: IBM Corp.). Continuous variables were summarized using mean and SD and tested with student *t*-tests. Not normally distributed variables were summarized with median and range and tested with Wilcoxon Signed Rank test. Paired tests were carried out for cases where both distal and proximal biopsies were available. Pearson correlation analysis was used to evaluate dependencies between variables. *p*-values < 0.05 were considered statistically significant.

## 3. Results

A total of 15 patients were included. Of the patients, the etiology was traumatic in three cases (3/15, 20%), congenital in three cases (3/15, 20%), idiopathic in two cases (2/15, 13%), tumor-related in six cases (6/15, 40%), and Möbius syndrome in one case (1/15, 7%). Four cases had incomplete facial nerve paresis (4/15, 17%) and eleven had complete facial palsy (11/15, 73%). The median House-Brackmann score was 6/6 (Range 4–6). Seven of the patients underwent direct neurotization of the original mimic muscles (7/15, 47%) and eight underwent CFNG in preparation for free muscle transplantation (8/15, 53%).

From the 15 patients, a total of 30 nerve biopsies were harvested. Of these, 22 specimens fulfilled quality criteria for axon quantification, diameter measurement, and fascicle count. Of the 22 specimens that met the quality standards, nine specimens were obtained proximal and thirteen specimens were obtained distal. The characteristics of the distal and proximal specimens are presented in Table 1.

**Figure 3:**
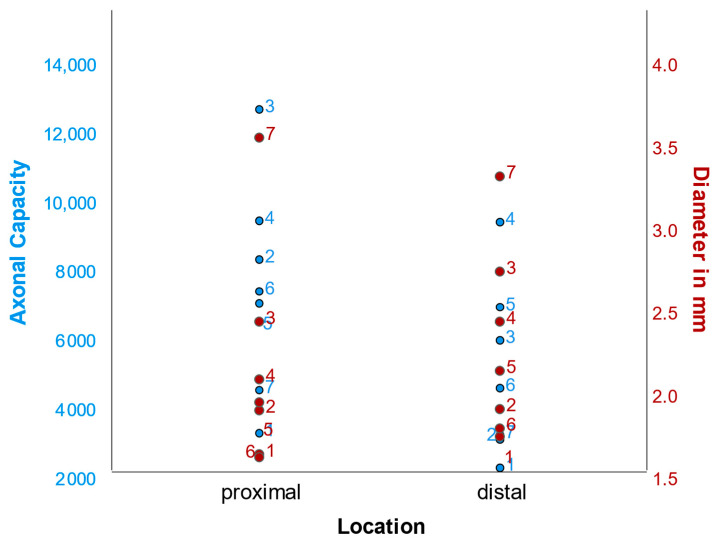
Plot of axonal capacity and diameter for patients in whom proximal and distal biopsies could be evaluated.

**Figure 4:**
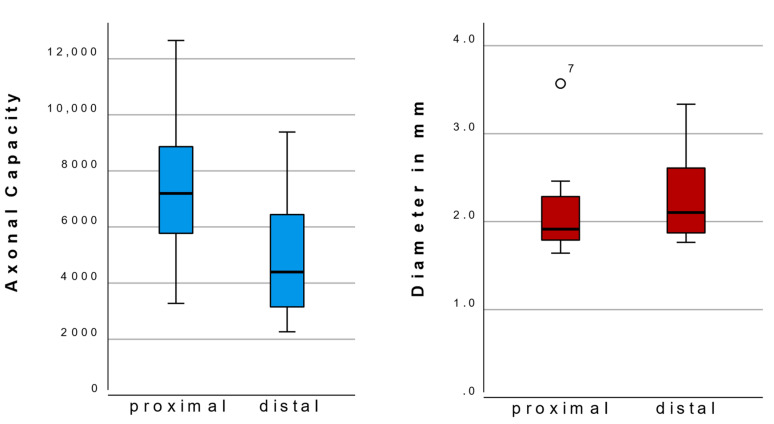
In those cases where both distal and proximal biopsies were available, a significant difference was seen for axonal capacity decrease towards the periphery (*p* = 0.047) but not for cross-sectional diameter (*p* = 0.136).

In seven patients, biopsies of the proximal and distal aspect of the sural nerve graft were available for analysis. Paired sample tests were performed showing a significant difference in fascicle number and axonal capacity (Table 2). Individual data for these cases are shown in Figure 3. A significant decrease in axonal capacity toward the periphery was observed, whereas the diameter tended to increase, although this was not significant (Figure 4).

The side of nerve harvest showed no significant differences related to nerve caliber and axonal capacity for proximal and distal biopsies (diameter: proximal *p* = 0.253, distal *p* = 0.506; axonal capacity: proximal *p* = 0.414, distal *p* = 0.922).

The median age of the operated patients was 37 years (5–62 years). Age did not correlate significantly with axonal capacity (proximal: R = −0.201, *p* = 0.603; distal: R = 0.317, *p* = 0.292).

## 4. Discussion

In all reanimation procedures, remarkably in CFNG, the axonal load plays a crucial role. To further investigate axon capacity in CFNG procedures, we compared our findings with relevant studies that provided data on donor and recipient nerves (Table 3) [30,31,32].

For cross-facial nerve grafts, a capacity greater than 900 donor axons was identified as a promising indicator for favorable outcomes in midfacial reanimation using free muscle grafts. Furthermore, Terzis et al. observed a correlation between donor axonal input and the axon numbers at the distal end of the nerve graft. However, no significant correlation was found for the axon counts at the distal end of the nerve graft and clinical outcomes [27].

Considering the intricate branching patterns of the facial nerve, which contribute to a wide range of facial expressions and functions, it is noteworthy to explore the potential of interfascicular division of the nerve graft. This division could facilitate extensive reanimation on the paralyzed side, enabling not only spontaneous smiling but also other symmetrical facial expressions [33].

Our findings revealed an average proximal axonal count of 6428 and a distal count of 3972 axons. Even the lowest measurement, which counted 1801 axons, could potentially yield more than 900 axons when equally divided in two.

Considering that the sural nerve offers significantly more axons compared to both the donor and recipient nerves, it could be deemed reasonable to divide the graft interfascicularly and connect multiple donor and recipient nerves, enabling a broader functional recovery. This approach takes into account axon capacity and aims to maximize functional regain.

The potential benefits of graft division are still a subject of debate, primarily due to concerns about potential interfascicular intermingling. Consequently, dividing the graft longitudinally could result in the disruption of endoneural SC-leading tubes, leading to inconsistent scaffolds for axonal outgrowth [25,34,35,36].

On the other hand, it is widely acknowledged that maintaining the fascicular structure is crucial for surgical repair of motor nerve injuries. Suturing the interfascicular epineurium to connect matching fascicles is recommended for nerves where the fascicular anatomy and somatotopy are clearly understood. In cases of direct nerve repairs, there is a consensus regarding the advantages of employing matching interfascicular sutures. However, for nerve grafting, particularly in the context of cable grafts such as CFNG, the advantages of interfascicular division remain a topic of ongoing debate [34].

Our study found that the side of nerve harvest showed no significant differences related to nerve caliber and axonal capacity for proximal and distal biopsies. This encourages the suitability of the sural nerve for CFNG, making it possible to harvest the sural nerve from both sides if more nerve is needed or one side shows anatomical varieties making harvesting inappropriate. This is in agreement with a study which investigated several nerve branches, finding no significant difference between the right and left sides of the nerve samples for the nerve area, fascicle area, number of fascicles, and average number of axons [32]. Our results demonstrated that the sural nerve has a relatively similar diameter proximally and distally, but a significantly higher axonal capacity and lower number of fascicles proximally. It is well known that nerves towards the periphery are less densely packed and have more fascicles. Whether the more perineural connective tissue can also serve as axon guidance structure or only the original fascicles is uncertain.

Our findings also showed that age did not correlate significantly with axonal capacity. Despite this result, Weiss et al. stated that the outcome of CFNG-driven gracilis free muscle flaps is age-related [12]. This finding can be explained due to different age-associated behavior in axonal regeneration [12,22]. Therefore, further investigations are needed to ultimately determine the influence of axonal load and patients’ age.

Different procedures have been described to sustain sufficient axonal load at the distal coaptation site. These include end-to-side neurorrhaphy with sensory axons to counteract SC senescence and intra- and post-operative electrostimulation [26,37,38,39].

Whilst non-facial donor nerves can provide greater axonal loads than cross-facial nerve grafts, muscle tone for symmetry at rest can be provided by facial donor nerve branches [30,40,41,42].

Looking at the commonly used nerve-to-masseter and cross-facial nerve graft, we agree that cross-facial nerve grafts can provide spontaneity in emotional expression and symmetry in resting tone but less excursion than nerve-to-masseter. Moreover, cross-facial nerve grafts require more recovery time than nerve-to-masseter transfers [43,44,45,46,47]. Even so, some studies suggest that spontaneity can also be accomplished using non-facial donor sources [48]. Considering the data and knowledge we have about CFNG and facial reanimation to this date, the findings of our study propose promising suitability of the sural nerve for CFNG [27].

Suitability and selection of donor nerves for neurotization is a crucial consideration, including several factors besides axon capacity. The location and accessibility, as well as the nature of the nerve injury and the specific goals of the reanimation surgery, influence donor nerve selection [47,49].

Sensory nerves are frequently used for CFNG because their loss does not significantly impact overall sensation and muscle function due to their functional redundancy. Such sensory nerves include the sural nerve, which is harvested from the lateral lower leg, or the lateral and medial antebrachial cutaneous nerve [50,51,52].

Axon counts for the lateral and medial antebrachial cutaneous nerve have just been published looking at donor and recipient nerve axon counts in gender-affirming radial forearm phalloplasty. The mean axon counts for the lateral antebrachial cutaneous nerve were 6957 ± 1098, and for the medial antebrachial cutaneous nerve they were 1866 ± 590 [53]. From the sheer axon numbers, those nerves could also be used for autologous nerve grafting [35].

Gyori et al. conducted a study on the axon numbers and landmarks of trigeminal donor nerves for corneal neurotization. Through dissection of non-embalmed cadavers, they found that the mean donor axon counts were 3146 ± 1069.9 for the supratrochlear nerve and 1882 ± 903 for the supraorbital nerve. Consequently, the supratrochlear and supraorbital nerves, both sensory nerves, can be considered potent donor nerves for ocular neuropathologies [54].

If the goal of neurotization is restoring motor function, however, then motor nerves may be used. They are commonly chosen based on their anatomical proximity to the recipient site and their functional resemblance to the damaged nerve. Frequently used motor nerves include the ulnar and radial nerve and the accessory nerve [44,49,55,56].

Where there is no suitable donor nerve available near the injury, nerve grafts may be used. Nerve grafts also apply when the injury cannot be bridged otherwise, and they can either be autografts, meaning they originate from the patient itself, or they can be allografts, which are harvested from a deceased donor [49].

Nerve growth factor (NGF) has been shown to play an essential role in nerve regeneration, promoting neuroprotective repair, survival, and differentiation of neurons [19,57,58]. Among other neurotrophins, brain-derived neurotrophic factor, and glial-derived neurotrophic factor (GDNF), along with their receptors and adhesion molecules, undergo increased activity in anticipation of axonal regeneration [19,59,60,61].

Additionally, it is important to look at various cell types crucial in nerve regeneration [19]. Here, specifically SC will be addressed as they fulfill a pivotal function in nerve regeneration. They form the myelin sheath around peripheral nerves and support their fiber outgrowth by providing a structural scaffold and producing various growth factors. Additionally, they establish an extracellular matrix that promotes axonal regrowth and interact with other cell types in the process of regeneration. An interesting illustration of this phenomenon occurs through SC encounter with fibroblasts, particularly at the site of injury after a complete severance. This interaction induces migratory behavior, which is crucial in guiding the regenerating axons across the wound site [36,62].

Considering this multifaceted contribution of many factors and cell types in nerve regeneration, existing studies on nerve injury and regeneration give compelling evidence indicating that chronic denervation of SC, insufficient neuronal plasticity, and misguidance of sprouting axons into incorrect nerve channels are the primary risk factors for inadequate functional recovery. This applies to nerve regeneration in general, and further investigation should particularly consider CFNG [19,27,60].

Across various medical fields, increasing understanding of the genetic landscape and cellular pathways holds the potential to facilitate the development of more precise therapies. This can lead to a reduction in off-site effects while enhancing the effectiveness of targeted treatments [63].

Another aspect in which CFNG can be enhanced concerns addressing the potential ramifications of size mismatch in nerve transplantation, as it can significantly impact the regenerative process. When the graft diameter is larger than the one from the recipient nerve, excessive scar tissue formation, compression, and impaired axonal growth can occur, resulting in poor functional recovery [64]. A graft of too small diameter may not provide a sufficient scaffold for regenerating axons, leading them to a failure of bridging the injury adequately. This aligns with our findings, suggesting a significant decrease in axonal capacity towards the periphery.

Size mismatch can also disrupt the alignment and orientation of nerve fibers, impeding the formation of proper connections and restoring both sensory and motor functions. Consequently, these mismatches can induce nerve sprouting, resulting in synkinesis extending to other areas besides the reinnervated region [65].

A comparison between our findings on the diameter of the sural nerve with donor and recipient nerve branches may provide insight into possible size. For the frontal branch of the facial nerve, an average cross-sectional diameter of 1.01 ± 0.26 was found [30]. Revising the extracranial course of the facial nerve, a 1.002 ± 0.4598 mm diameter for the zygomatic branch and a 0.99 ± 0.3962 mm diameter for the buccal branch of the facial nerve have both been published [66]. Our findings showed a mean proximal diameter of 2.15 ± 0.59 mm (n = 9) and a mean distal diameter of 2.16 ± 0.51 mm (n = 13) for the sural nerve. Therefore, techniques to optimize size match in CFNG should be further explored.

Future studies should investigate the genetic profile of donor nerves in order to achieve sufficient and more successful results in nerve reanimation surgery, since several genes have been associated with nerve regeneration following nerve transplantation [19].

## 5. Conclusions

Our analysis of the microanatomy of the sural nerve could facilitate its routine clinical use for CFNG procedures and facial reanimation. Overall, this study may provide a more panoramic view of the microscopic characteristics of the sural nerve and guide targeted therapy for FP patients.

## 6. Limitations

This study is not without limitations. The samples taken proximally failed quality control in 6/15 cases. That rate is significantly higher compared to the distally obtained biopsies (6/15 vs. 2/15). We attribute this difference to the greater density of axons and fascicles, which could not be adequately processed by the fixative solution and staining solution.

Our sample size might be too small for a clear statement of statistical significance. However, there are recent studies in facial palsy research that provide novel insights into facial palsy therapy based on comparable sample sizes [67]. While our study revealed novel insights, further research and larger studies are needed for reliable statistical significance and clinical outcomes.

## Figures and Tables

**Table 1:** Histomorphometric characteristics of distal and proximal obtained specimens.

	Proximal (n = 9)	Distal (n = 13)
Fascicle count	5 (3–11) ^1^	9 (5–12) ^1^
Diameter (mm)	2.15 ± 0.59	2.16 ± 0.51
Axonal capacity	6428 ± 3277	3972 ± 2143

^1^ Median (Min–Max).

**Table 2:** Paired tests between proximal and distal biopsy sampling.

	Proximal (n = 7)	Distal (n = 7)	*p*-Value
Fascicle count	4 (3–7) ^1^	7 (5–11) ^1^	0.017 ^2^
Diameter (mm)	2.13 ± 0.66	2.25 ± 0.57	0.136
Axonal capacity	7324 ± 3105	4872 ± 2528	0.047

^1^ Median (Min–Max), ^2^ Wilcoxon Signed Rank Test.

**Table 3:** Comparison of previous data from relevant studies and our findings of axonal capacity of the sural nerve.

Donor and Recipient Nerve Branches	Axonal Capacity, Mean ± SD	Sural Nerve, Our Findings	Axonal Capacity, Mean ± SD
Frontal branch, facial nerve	1191 ± 668 [30]	proximal, n = 7	7324 ± 3105
Zygomatic branch, facial nerve	3199 ± 1864 (r), 3338 ± 228 (l) [32]	distal, n = 13	3972 ± 2143
Buccal branch, facial nerve	2386 ± 1368 (r), 2344 ± 1115 (l) [32]		
Gracilis branch, obturator nerve	598 ± 83 [31]		

## Data Availability

All data were presented in this paper. Inquiries regarding the raw data can be addressed to the corresponding author at any time.
